# Genetic variants in lncRNA SRA and risk of breast cancer

**DOI:** 10.18632/oncotarget.7995

**Published:** 2016-03-08

**Authors:** Rui Yan, Kaijuan Wang, Rui Peng, Shuaibing Wang, Jingjing Cao, Peng Wang, Chunhua Song

**Affiliations:** ^1^ Department of Epidemiology and Statistics, College of Public Health, Zhengzhou University, Zhengzhou, 450001, PR China; ^2^ Department of Tumor Epidemiology, Henan Key Laboratory of Tumor Epidemiology, Zhengzhou, 450001, PR China

**Keywords:** breast cancer, SRA, lncRNA, genetic susceptibility, interaction

## Abstract

Long non-coding RNA (lncRNA) steroid receptor RNA activator (SRA) has been identified to activate steroid receptor transcriptional activity and participate in tumor pathogenesis. This case-control study evaluated the association between two haplotype tagging SNPs (htSNPs) (rs10463297, rs801460) of the whole SRA sequence and breast cancer risk. We found that rs10463297 TC genotype significantly increased BC risk compared with CC genotype in both the codominant (TC vs. TT: OR=1.43, 95 % CI=1.02–2.00) and recessive (TC+CC vs. TT: OR=1.39, 95 % CI=1.01–1.92) genetic models. Both TC, TC + CC genotypes of rs10463297 and GA, AA, GA+AA genotypes of rs801460 were significantly associated with estrogen receptor (ER) positivity status. rs10463297 TC (2.09 ± 0.41), CC (2.42 ± 0.51) and TC + CC (2.20 ± 0.47) genotypes were associated with higher blood plasma SRA mRNA levels compared with the TT genotype(1.45 ± 0.34). Gene–reproductive interaction analysis presented a best model consisted of four factors (rs10463297, age, post-menopausal, No. of pregnancy), which could increase the BC risk with 1.58-fold (OR=1.58, 95 % CI=1.23–2.03). These findings suggest that SRA genetic variants may contribute to BC risk and have apparent interaction with reproductive factors in BC progression.

## INTRODUCTION

Breast cancer is the most frequently diagnosed malignant tumor and the first leading cause of cancer death among females [1, 2]. A large number of reproductive factors have been reported to be associated with BC, including early menarche, late menopause, no breast-feeding history for born baby, nullparity, abortion and family history of BC [3]. Moreover, a series of susceptibility genes have been identified to be implicated with breast cancer risk, and the association between single nucleotide polymorphisms (SNPs) and risk of BC has been reported [4, 5]. It is generally considered that genetic susceptibility, reproductive factors and gene–reproductive factors interactions all contribute to the development of BC.

Up to 98% of the transcriptional output of the human genome could represent RNA that do not code for protein [6]. These ‘non-coding RNAs' (ncRNAs) were previously believed to be transcriptional noise, but now accumulating evidences suggest that they play important roles in cell proliferation, differentiation, apoptosis, metabolism and immune [7]. A basic classification criterion of ncRNAs is based on their length: small ncRNAs and long ncRNAs (lncRNAs). Small ncRNAs are processed from longer precursors [8]. Over the past few years, a wealth of studies have highlighted the importance of small ncRNAs, especially microRNAs (miRNAs), in the development of cancers, and their variants were associated with various cancer risks [9–11]. By contrast, lncRNAs are eukaryotic RNAs longer than 200 nucleotides, lacking open reading frame, having no protein coding capacity, and function without major prior processing [12]. Recent studies have indicated that lncRNAs may play regulatory and structural roles through diverse molecular mechanisms in important biological processes [13]. LncRNAs contribute to carcinogenesis, and deliver functions in controlling cell cycle progression, apoptosis, invasion, and migration. Several studies have highlighted the importance of lncRNA and their genetic variants in the development of cancers. For example, H19 is an estrogen-inducible gene and plays a key role in cell survival, which may serve as a biomarker for breast cancer diagnosis and progression [14], and a significantly decreased risk of bladder cancer was found for H19 rs2839698 TC carriers [15]. rs11752942 AG+GG in the lincRNA-uc003opf.1 exon had a significantly reduced risk of esophageal squamous cell carcinoma (ESCC), the rs11752942G allele could markedly attenuate the level of lincRNA-uc003opf.1 and affect cell proliferation and tumor growth [16]. HOTAIR has been widely identified to participate in tumor pathogenesis, acting as a promoter in colorectal cancer carcinogenesis, and rs7958904 CC decreased the risk of colorectal cancer compared with GG genotype [17]. Li et al., founded that the C to T base change at rs12325489 could disrupts the binding site for miRNA-370, influencing lincRNA-ENST00000515084 transcriptional activity and affecting breast cancer cell proliferation and tumor growth [18].

Another lncRNA that may play an important role in breast cancer is the steroid receptor RNA activator (SRA). SRA, located on chromosome 5q31.3 and containing five exons and four introns, was initially characterized as belonging to the growing family of functional non-coding RNAs, specifically activating steroid receptor transcriptional activity [19]. The level of SRA is increased in breast tumors and the expression of SRA correlates with estrogen receptor (ER) and progesterone receptor (PR) levels, which may alter ER/PR action and promote tumorigenesis [20]. However, to date, no research has been executed to evaluate the SRA polymorphism and the risk of BC. On the basis of the above description, we hypothesized that functional SNPs in SRA might have association with the BC risk. Tagging SNPs of SRA were selected with the Haploview version 4.2 software. Four particular SNPs (rs10463297, rs801460, rs250425 and rs250426) were representative and could capture all the other common SNPs with a tagging threshold of r^2^ > 0.80. However, rs250425 was not in the region of SRA and the refSNP alleles of rs250426 was A/G/T (FWD) according to the NCBI dbSNP database, and we could not find a restriction enzyme to cut the PCR amplification products and genotyping accurately. According to the HapMap data of Chinese Han populations in Beijing, T and C allele frequency of SRA rs10463297 were 0.467 and 0.533 respectively. C and T allele frequency of SRA rs801460 were 0.412 and 0.588 respectively. So we finally selected these two particular SNPs (rs10463297 and rs801460) for our study by using the criteria of a minor allele frequency (MAF) ≥0.1 in the Chinese Han population. We genotyped the two SRA haplotype tagging SNPs (rs10463297 and rs801460) in a population-based case–control study comprising 489 BC patients and 495 age frequency matched controls from China. The association between the SRA SNPs and breast cancer risk were investigated by molecular epidemiology.

## RESULTS

### Characteristics of the study population

The baseline characteristics of the 489 BC cases and 490 cancer-free controls are shown in Table 1. The mean age was 48.45±10.13 and 49.14±10.06 years for BC cases and healthy controls, respectively. As expected, the mean age for two groups paired quite well. There was no significant differences between case and control groups with respect to other baseline characteristic factors, including age at menarche and menopause, menstrual history, No. of abortion, breast-feeding and family history.

**Table 1:** Characteristics of breast cancer cases and cancer-free controls

Characteristics	Cases (%)^a^	Controls (%)^a^	*P*	OR(95%CI)
	(n=489)	(n=495)		
Age (Mean ± SD)	48.45±10.13	49.14±10.06	0.28^b^	
Age at menarche (Mean ± SD)	14.37±1.68	14.19±1.63	0.10^b^	
Age at menopause				
≤50	144(72.00)	146(71.22)		1
>50	56(28.00)	59(28.78)	0.86^b^	0.96(0.63, 1.48)
Menstrual history				
Pre-menopausal	289(59.10)	290(58.59)		1
Post-menopausal	200(40.90)	205(41.41)	0.96^c^	0.98(0.76, 1.26)
No. of pregnancy				
0-2	230(47.03)	254(51.31)		1
≥3	259(52.97)	241(48.69)	0.18^c^	1.19(0.92, 1.52)
No. of abortion				
0-2	430(87.93)	447(90.30)		
≥3	59(12.07)	48(9.70)	0.23^c^	1.28(0.85, 1.91)
Breast-feeding				
No	25(94.89)	32(6.46)		
Yes	464(5.11)	463(93.54)	0.36^c^	1.28(0.75, 2.20)
Family history of BC				
No	470(96.11)	483(97.58)		
Yes	19(3.89)	11(2.42)	0.13^c^	1.78(0.84, 3.77)

a The absolute number (n) and percentage (%) of cases and controls.

b Student's t test

c Two-sided χ^2^ test, *P*<0.05 was considered statistically significant.

### Associations between SRA genotypes and the risk of BC

The genotype and allele distributions of two SNPs (rs10463297 and rs801460) in cases and controls are shown in Table 2. The observed genotype frequencies for the two SNPs agreed with the expected ones from the Hardy–Weinberg equilibrium in the 495 cancer-free controls, respectively (P = 0.14 for rs10463297, P = 0.06 for rs801460). No significant difference was observed in the frequency distribution of rs801460 polymorphisms between breast cancer patients and healthy controls. Individuals with TC (P=0.04, OR=1.43, 95 %CI=1.02–2.00) and TC + CC (P=0.04, OR=1.39, 95 %CI=1.01–1.92) genotype of rs10463297 showed increased risk to BC compared with TT genotype. After adjustment for age, menopausal status, number of pregnancies and abortions, breast-feeding status and family history of BC in fist-degree relatives, the association were still significant for individuals with TC (P=0.03, adjusted OR=1.47, 95 %CI=1.04–2.10, P=0.03) and TC + CC (P=0.04, adjusted OR=1.39, 95 %CI=1.02–1.95) genotype of rs10463297.

**Table 2:** Genotype among cases and controls and their association with BC risk

Genotype	Cases (%)	Controls (%)	*P*^a^	P^b^	OR(95% CI)^b^	*P*^c^	OR(95% CI)^c^
	(n=489)	(n=495)					
rs10463279							
TT	78(15.95)	103(20.81)			1		1
TC	284(58.08)	263(53.13)		**0.04**	**1.43(1.02, 2.00)**	**0.03**	**1.47(1.04, 2.10)**
CC	127(25.97)	129(26.06)	**0.14**	0.18	1.30(0.89, 1.90)	0.20	1.29(0.88, 1.91)
TC+CC	411(89.05)	392(79.19)		**0.04**	**1.39(1.01, 1.92)**	**0.04**	**1.39(1.02, 1.95)**
T	440	469			1		
C	538	521		0.29	1.10(0.92, 1.31)		
rs801460							
GG	97(19.84)	91(18.38)			1		1
GA	272(55.62)	266(53.74)		0.81	0.96(0.69, 1.34)	0.82	1.04(0.74, 1.47)
AA	120(24.54)	138(27.88)	***0.06***	0.29	0.82(0.56, 1.19)	0.39	0.84(0.58, 1.24)
GA+AA	392(80.16)	404(81.62)		0.56	0.91(0.66, 1.25)	0.86	0.97(0.70, 1.34)
G	466	448			1		
A	512	542		0.29	0.91(0.76, 1.08)		

a *P* value of Hardy–Weinberg equilibrium in controls

b Chi square test for genotype distributions between cases and controls

c Data were calculated by logistic regression analysis with adjusted for age, age at menarche, menopausal status, number of pregnancy and abortion, breast-feeding status, family history of BC in first-degree relatives

### Functional relevance of rs10463297 genotypes on SRA mRNA expression

**Figure 1:**
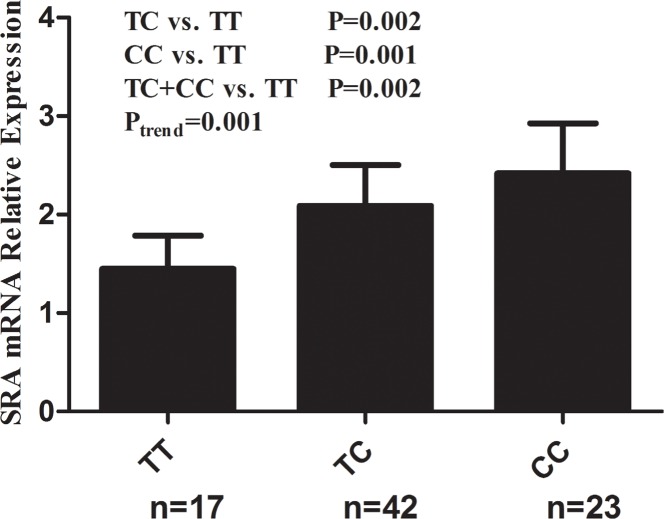
Correlation between rs10463297 genotypes and SRA mRNA relative expression Relative SRA mRNA expression levels in blood plasma from 82 cancer-free controls were significantly higher for the TC (2.09 ± 0.41), CC (2.42 ± 0.51) and TC + CC genotypes (2.20 ± 0.47) than the TT genotype (1.45 ± 0.34) (P = 0.002, 0.001 and 0.002, respectively).

We further randomly selected 82 cancer-free controls and investigated the correlations between rs10463297 genotypes and SRA mRNA expression level in blood plasma. Among the 82 cancer-free controls, 17 had TT genotype of rs10463297, 42 had TC genotype of rs10463297, and 23 had CC genotype of rs10463297. As shown in Figure 1, SRA mRNA expression levels were significantly higher for the TC (2.09 ± 0.41), CC (2.42 ± 0.51) and TC + CC genotypes (2.20 ± 0.47) than the TT genotype (1.45 ± 0.34) (P = 0.002, 0.001 and 0.002, respectively). A significance increased SRA mRNA expression towards was found for the effect of the C allele (P_trend_=0.001).

### Haplotype analyses and combined effect of two SNPs

Haplotype analysis was performed to evaluate the combined effect of the two polymorphisms on the risk of BC. A total of four haplotypes were derived from the observed genotypes (Table 3), of which C_rs10463297_ A_rs801460_ was the most common haplotype in cases and controls. No significant association with BC risk was observed for these four haplotypes. We further calculated the joint effect and potential locus-locus interaction on BC risk by categorizing the SNPs (rs10463297 and rs801460) into the number of combined variant alleles. When compared to individuals with 0-1 mutation allele, no statistical increased risk for BC in each subgroup and no increased dose-dependent manner was observed on the combined effect of the two SNPs (Table 4).

**Table 3:** Haplotype analysis of rs10463297and rs801460 polymorphism sites in SRA

Haplotype^a^	Cases (%)	Controls (%)	χ^2^	*P*	OR (95%CI)
C A	437.76(44.8)	443.17(44.8)	0.00	0.99	1.00(0.84, 1.19)
C G	100.24(10.2)	77.83(7.9)	3.41	0.06	1.34(0.98, 1.83)
T A	74.24(7.6)	98.83(10.0)	3.51	0.06	0.74(0.54, 1.02)
T G	365.76(37.4)	370.17(37.4)	0.99	0.914	1.00 (0.83, 1.20)

a SNPs sequence: rs10463297and rs801460

**Table 4:** Combined effect of the two SNPs on breast cancer

Combined SNPs ^a^	Cases (%)	Controls (%)	χ^2^	*P*	OR (95%CI)
0	58(11.86)	67(13.53)			1
1	51(10.23)	48(9.70)	0.58	0.45	1.23(0.72, 2.08)
2	216(44.17)	209(42.22)	0.76	0.39	1.19(0.80, 0.78)
3	89(18.20)	87(17.58)	0.51	0.48	1.19(0.75, 1.87)
4	75(15.34)	84(16.97)	0.02	0.90	1.03(0.65, 1.65)
			0.00^b^	0.99^b^	
Total	489	495			

a The SNPs: rs10463297, rs801460

b Trend Chi-square and P values

### Stratified analysis of SNP genotypes and BC risk

A stratified analysis assessing the associations between the SRA SNP genotypes and the risk of breast cancer was conducted. As indicated in Table 5, we found that the increased risk of breast cancer associated with the rs10463297 variant allele was significant among age >50 (P=0.03, adjusted OR =1.79, 95% CI=1.05-3.05). No significant association with SRA polymorphisms was observed in other subgroups.

**Table 5:** Stratification analysis of the three SNPs polymorphisms and BC susceptibility

	rs10463279 (TT/TC+CC)			*P*	rs801460(GG/GA+AA)			*P*
Variables	Case	Control	OR (95%CI)^a^		Case	Control	OR (95%CI)^a^	
Age								
≤50	50/255	56/244	1.25(0.81, 1.93)	0.32	62/243	55/245	0.98(0.65, 1.34)	0.94
>50	28/156	47/148	**1.79(1.05, 3.05)**	**0.03**	35/149	36/159	1.04(0.61, 1.77)	0.90
Age at menarche								
≤14	51/254	68/261	1.33(0.81, 2.01)	0.18	66/239	57/272	0.82(0.54, 1.23)	0.32
>14	27/157	35/131	1.51(0.85, 2.67)	0.16	31/153	34/132	1.33(0.76, 2.33)	0.33
Age at menopause								
≤50	22/122	32/166	1.53(0.82, 2.85)	0.19	27/117	27/121	1.04(0.56, 1.94)	0.90
>50	8/48	15/47	1.79(0.66, 4.82)	0.25	13/43	11/51	0.67(0.25, 1.77)	0.42
Menopause								
Pre-menopausal	48/241	57/233	1.27(0.82, 1.97)	0.28	57/232	53/237	0.98(0.63, 1.50)	0.91
Post-menopausal	30/170	46/159	1.63(0.96, 2.75)	0.07	40/160	38/167	0.95(0.57, 1.59)	0.85
No. of pregnancy								
≤2	34/196	54/200	1.47(0.91, 2.39)	0.12	42/188	45/209	0.94(0.58, 1.52)	0.81
>2	44/215	49/192	1.22(0.77, 1.93)	0.40	55/204	46/195	0.85(0.54, 1.32)	0.46
No. of abortion								
≤2	67/363	95/352	1.46(0.93, 2.07)	0.06	84/346	85/362	0.98(0.70, 1.38)	0.93
>2	11/48	8/40	0.74(0.26, 2.13)	0.57	13/46	6/42	0.47(0.15, 1.53)	0.21
Breast-feeding								
No	5/20	7/25	0.86(0.21, 3.58)	0.84	3/22	7/25	1.67(0.33, 8.41)	0.54
Yes	73/391	96/367	1.43(0.94, 2.01)	0.06	94/370	84/379	0.93(0.67, 1.30)	0.67
Family history								
No	77/393	101/383	1.37(0.98, 1.91)	0.07	92/378	90/394	1.01(0.72, 1.40)	0.97
Yes	1/18	2/9	2.41(0.14, 4.17)	0.54	5/14	1/10	0.29(0.02, 4.69)	0.38

a Data were calculated by logistic regression analysis with adjusted for age, age at menarche, menopausal status, number of pregnancy and abortion, breast-feeding status, family history of BC in first-degree relatives (the stratified factor in each stratum excluded)

### Receptor status and BC risk

We further demonstrated the association of rs10463297 and rs801460 polymorphism genotypes with the clinicopathological features in Table 6, including ER status, PR status and HER-2 status. Among the 489 cases with immunohistochemistry records of tumor tissues, 339 (69.39 %) cases were ER positive, 283 (57.87%) cases were PR positive, and 327 (66.87 %) cases were HER-2 positive. In the case only analysis, compared with TT genotype of SNP rs10463297, TC (P=0.001, adjusted OR=2.45, 95 % CI=1.44–4.17) and TC + CC (P=0.002, adjusted OR=2.24, 95 % CI= 1.34–3.69) genotype were associated with ER positivity. Similarly, rs801460 GA (P=0.01, adjusted OR=1.92, 95 % CI=1.17–3.14), AA (P=0.01, adjusted OR=2.25, 95 % CI=1.23–4.13) and GA + AA (P=0.004, adjusted OR=1.99, 95 % CI=1.25–3.19) genotype were associated with ER positivity in BC patients. No significant association was found between SRA variants and PR, HER-2 status.

**Table 6:** The associations between two SNPs and ER, PR and HER-2 status of breast cancer patients

Genotype	ER		*P*^a^	OR (95%CI)^a^	PR		*P*^a^	OR (95%CI)^a^	HER-2		*P*^a^	OR (95%CI)^a^
	Positive (n=339) %	Negative (n=150) %			Positive (n=283) %	Negative (n=206) %			Positive (n=327) %	Negative (n=162) %		
rs10463297												
TT	42(12.39)	36(2.45)		1	42(14.84)	36(17.48)		1	51(15.60)	27(16.67)		1
TC	212(62.54)	72(48.00)	**0.001**	**2.45(1.44, 4.17)**	171(60.43)	113(54.85)	0.28	1.33(0.80, 2.22)	192(58.72)	92(56.79)	0.63	1.14(0.67, 1.97)
CC	85(25.07)	42(28.00)	0.05	0.99 (0.95, 1.04)	70(24.73)	57(27.67)	0.75	1.10(0.61, 1.97)	84(25.69)	43(26.54)	0.97	1.01(0.55, 1.87)
TC+CC	297(87.61)	114(76.00)	**0.002**	**2.24(1.34, 3.69)**	241(85.16)	170(82.52)	0.37	1.26 (0.77, 2.06)	276(84.40)	135(83.33)	0.78	1.08(0.64, 1.81)
rs801460												
GG	56(16.52)	41(27.33)		1	56(19.79)	41(19.90)		1	63(19.27)	34(20.99)		1
GA	195(57.52)	77(51.33)	**0.01**	**1.92(1.17, 3.14)**	157(55.48)	115(55.83)	0.96	1.01(0.63, 1.63)	180(55.04)	92(56.79)	0.72	1.10(0.67, 1.80)
AA	88(25.96)	32(21.34)	**0.01**	**2.25(1.23, 4.13)**	70(24.73)	50(24.27)	0.77	1.09 (0.62, 1.90)	84(25.69)	36(22.22)	0.29	1.38(0.76, 2.52)
GA+AA	283(83.48)	109(72.67)	**0.004**	**1.99(1.25, 3.19)**	227(80.21)	165(80.10)	0.84	1.00(0.98, 1.03)	264(80.73)	128(79.01)	0.26	1.02(0.98, 1.05)

a Adjusted for age, age at menarche, menopausal status, number of pregnancy and abortion, breast-feeding status, family history of BC in first-degree relative

### Gene–reproductive factors interaction analysis

MDR analysis was performed to analyze the gene–reproductive factors interaction with two SNPs (rs10463297 and rs801460), age, the ages of menarche and menopause, menopausal status, number of pregnancies and abortions, breast-feeding and family history of BC in fist-degree relatives (Table 7). The best model consisted of four factors (rs10463297, age, post-menopausal, No. of pregnancy) with TBA: 0.56 and CVC: 3/10, which could categorize the BC risk in the “high-risk group” 1.58-fold (P<0.001, OR=1.58, 95 % CI=1.23–2.03) compared to the “low-risk group”.

**Table 7:** Interaction results between the SRA SNPs and reproductive factors by MDR

Model	TBA^a^	CVC^b^	χ^2^	OR(95%CI)	*P*
rs10463279	0.5257	6/10	3.87	***1.38 (1.01, 1.92)***	**0.049**
Post-menopausal, No. of pregnancy	0.5411	8/10	6.47	***1.39(1.08, 1.78)***	**0.011**
Post-menopausal, No. of pregnancy, Breast-feeding	0.5493	6/10	8.57	***1.46 (1.13, 1.87)***	**0.003**
rs10463279, Age, Post-menopausal, No. of pregnancy	0.5601	3/10	12.58	***1.58(1.23, 2.03)***	**<0.001**

a Testing balance accuracy

b Cross-validation consistency

### FPRP values for all significant associations

Moreover, for all the significant associations observed above, we calculated the false positive report probability (FPRP) values to test whether there were false positive associations. As shown in Table 8, when we set the assumption of prior probability at 0.25, all of the significant associations were noteworthy (FPRP <0.5). After correction for the assumption of prior probability (p=0.10), the rs10463297 TC with BC and ER (FPRP=0.351 and 0.196 respectively), rs10463297 TC+TT with BC and ER (FPRP=0.378 and 0.194 respectively), rs801460 GA, AA and GA+AA with ER (FPRP=0.341, 0.456 and 0.242 respectively) were still noteworthy.

**Table 8:** FPRP values for associations between BC risk, ER and genotypes in stratified factors

Genotype		Stratified factors	BC/ER	Positive OR (95%CI)	P^c^	Prior probability			
						0.25	0.1	0.01	0.001
rs10463279	TT/TC	All subjects	BC	1.43(1.02, 2.00)^a^	0.04^a^	**0.153**	**0.351**	0.856	0.984
				1.47(1.04, 2.10)^b^	0.03^b^	**0.159**	**0.362**	0.862	0.984
	TT/TC+TT	All subjects	BC	1.39(1.00, 1.92)^a^	0.05^a^	**0.168**	**0.378**	0.870	0.985
				1.39(1.02, 1.95)^b^	0.04^b^	**0.355**	0.623	0.948	0.995
	TT/TC+CC	Age>50	BC	1.79(1.05, 3.05)^b^	0.03^b^	**0.273**	0.530	0.925	0.992
rs10463279	TT/TC	All cases	ER	2.45(1.44, 4.17)^b^	0.001^b^	**0.075**	**0.196**	0.729	0.964
	TT/TC+CC	All cases	ER	2.24(1.34, 3.69)^b^	0.002^b^	**0.074**	**0.194**	0.726	0.964
rs801460	GG/GA	All cases	ER	1.92(1.17, 3.14)^b^	0.01^b^	**0.147**	**0.341**	0.850	0.983
	GG/ AA	All cases	ER	2.25(1.23, 4.13)^b^	0.01^b^	**0.218**	**0.456**	0.902	0.989
	GG/GA+AA	All cases	ER	1.99(1.25, 3.19)^b^	0.004^b^	**0.096**	**0.242**	0.778	0.973

a The crude OR and P value.

b The adjusted OR and P value in the logistic regression analysis.

## DISCUSSION

Single nucleotide polymorphisms (SNPs) have been confirmed to have profound effects on gene expression and function, and participate in carcinogenesis. Recently, studies on the effects of SNPs have extended to functional lncRNAs. SNPs in several lncRNAs have been reported to be associated with cancer risk. In this population-based case–control study in a Chinese population, we selected htSNPs in lncRNA SRA region, and assessed the association between these genetic variants and breast cancer susceptibility. Our results shown rs10463279 statistically significant associated with ER positivity status, increased breast cancer risk and elevated SRA mRNA expression, and it may have interaction with reproductive factors in BC progression.

Previous studies have indicated that lncRNAs genetic variation might change the lncRNA structure, influence the level of lncRNA expression and contribute to carcinogenesis. In our study, we found TC genotype of SRA rs10463279 polymorphism was associated with significantly increased risks of BC susceptibility in both the codominant (TC vs. TT: P=0.04, OR=1.43, 95 %CI=1.02–2.00) and recessive (TC+CC vs. TT: P=0.04, OR=1.39, 95 %CI=1.01–1.92) inheritance genetic models. On analyzing the data in SRA polymorphism with logistic regression, TC+CC genotype of rs10463279 was associated with the risk of BC in women of age>50 (P=0.03, adjusted OR =1.79, 95% CI=1.05-3.05). Further functional studies are required to validate whether SRA polymorphisms might affect the lncRNA structure have interaction with micro-RNA for breast cancer susceptibility, metastases, and prognosis.

The results of molecular epidemiology studies were always accompanied by high probability of false positive [21–23]. The false positive report probability (FPRP) calculation was aimed to report the true association between the genetic variant and the disease, depends not only on the observed P value, but also on both the prior probability and the statistical power of the test [24]. We subsequently calculated the FPRP for all significant genetic effects observed in our study to test the false positive associations. The results of FPRP indicated that our results were less likely to be false positives, which implies the functional SNPs in SRA might be involved in the breast cancer development with a high likelihood.

The SRA RNA is a non-coding RNA that strongly associated with breast cancer and participate in nuclear coactivation for several hormone-related systems [25], including the estrogen receptor [19, 26, 27], androgen receptor [28], progesterone receptor [19, 29] and thyroid hormone receptor [30]. A study by Leygue et al., reported that SRA expression could correlate positively or negatively with ER and PR levels, depending on the subgroup considered [20]. In that study, SRA expression was similar in ER-/PR- and in ER+/PR+ tumors, and SRA expression in these two subgroups was significantly lower than that observed in ER-/PR+ and ER+/PR- tumors. In our study, we further estimated the association between SRA polymorphism and ER, PR and HER-2 in BC patients, to clarify the role of SRA polymorphism in the pathologic state of BC. No significant association was observed between PR, HER-2 status and the genetic variants. However, both rs10463297 TC, TC + CC and rs801460 GA, AA, GA+AA genotype were significantly associated with ER positivity, which is a novel finding and suggests that SRA polymorphisms might have potential effects on estrogen receptor in breast cancer development.

In the current study, SRA rs10463297 TC and TC+CC genotype were associated with increased BC risk in the Chinese population. Furthermore, in cancer-free controls, variant genotypes of rs10463297 were associated with increased serum mRNA expression levels of SRA, suggesting SRA polymorphism may have a potential functional impact on mRNA levels, thus supporting a role in the susceptibility to BC.

BC is a complex disease likely resulting from multiple interacting genetic polymorphisms and gene–reproductive factor interactions [31–33]. In this study, the gene–reproductive factor interaction on breast cancer susceptibility was examined by using a MDR method. A nominally significant interaction was found for rs10463297, age, post-menopausal, No. of pregnancy. One of the advantages of MDR method is that false-positive results due to multiple testing are minimized [34]. Thus, we can carefully suggest that a potential influence of age, post-menopausal, No. of pregnancy interaction with SRA polymorphisms rs10463297 contribute to the risk of BC in a central Chinese population.

This is the first study to our knowledge to examine the role of SRA genetic polymorphism in BC carcinogenesis and focus on the gene–reproductive factor interactions on BC risk in a Chinese women population. There were some strengths of this study that should be noted. First, our controls were selected from people in a large sampling survey based on community, not from hospital, which significantly diminished the effect of selection bias. Second, a well-defined cohort of newly pathological diagnosed cases avoided the prevalence-incidence bias. Third, the controls and the cases were matched on age, and the baseline characteristic distributions in our control group were similar to case group. Therefore, we believed that selection bias was not substantial and not likely to influence the analyses of our study. Furthermore, for all significant genetic effects observed in our study, we calculated the FPRP. It is proved that our results are less likely to be false positives according to the FPRP results. However, several limitations may exist in the present study. The sample size of our study was not large, and the statistical power of the study may be limited. Therefore, it will be worth-while to validate these findings in larger studies with other ethnic populations, and clarify the genetic mechanisms of the SRA in the etiology of BC.

In summary, our results reveal for the first time that a novel SNP rs10463297 located in SRA gene was significantly associated with increased risk of BC. SRA rs10463297 polymorphism might be a helpful genetic marker to predict BC predisposition. Larger prospective studies are needed to validate our findings and further investigations are required to understand the exact mechanisms of SRA rs10463297 polymorphism in BC cells.

## MATERIALS AND METHODS

### Subjects

All subjects participating in this study were genetically unrelated ethnic Chinese women. 489 newly diagnosed breast cancer patients with pathologically confirmed incident primary BC were recruited from the First Affiliated Hospital of Zhengzhou University and the Third Affiliated Hospital of Zhengzhou University between 2014 and 2015. At the same period, 495 healthy controls were randomly recruited from a pool of >20000 subjects participated community-based chronic diseases program of Henan province. All the controls were genetically unrelated with cases, free of any cancer, having no the history of chronic diseases and were frequency matched to the cancer patients on the basis of their age. Informed consent was obtained from each study participant. Reproductive variables including age of menarche, premenopausal or postmenopausal, age of menopause, number of pregnancy, number of abortion, breast-feeding history for born baby (yes, no), and family history of BC in first-degree relatives (yes, no) were obtained by a structured questionnaire through face-to-face interviews. Pathological data of BC patients, including of the estrogen receptor (ER), progesterone receptor (PR) and human epidermal growth factor receptor-2 (HER-2) status, were obtained by immunohistochemistry (IHC) from pathology reports.

The study was approved by the ethical review committee of Zhengzhou University Committee for Medical and Health Research Ethics.

### DNA extraction

For each participant, venous blood (5 ml) was collected into a test tube containing ethylene diamine tetra acetic acid (EDTA). Genomic DNA was extracted from peripheral blood samples of all participants using the DNA Extraction Kit of TIANGEN BIOTECH (Beijing) according to the manufacturer's instructions. The extracted DNA was stored at −80°C until use.

### SNP genotyping

The genotyping of rs10463297 was determined by polymerase chain reaction-restriction fragment-length polymorphism (PCR-RFLP), while SRA rs801460 was genotyped with created restriction site PCR (CRS-RFLP) assays.

The primers used for PCR amplification were designed by Primer 6.0 software (Table 9). PCR primers were further verified by NCBI BLAST (http://blast.ncbi.nlm.nih.gov/Blast.cgi/) to assess the possibility of amplifiation of any non-specifi DNA sequences and synthesized commercially. For each sample, PCR amplification was performed in a final volume of 30 μl, which contained 15 μl 2×Tap PCR MasterMix, 0.5 μl each primer (10 μM), 50 ng DNA, and 13 μl deionized water. Thermocycling conditions of PCR were as follows: initial denaturation at 95°C for 5 min, 35 cycles of PCR consisting of denaturation at 94°C for 30 s, optimal annealing temperature (Table 9) for 45 s and extension at 72°C for 45 s, and final extension step of 72°C for 5 min.

**Table 9:** PCR information of the two SNPs

SNP	Chr:position	MAF^a^	Genotyping assay	Annealing Tm(°C)	Primers
rs10463297 T>C	Chr5:140556654	C=0.467	PCR-RFLP	57.0	Sense: GGTGGCTCTCCTCTACTT Antisense: GTCCATTCTGTCTTCACTTAG
rs801460 G>A	Chr5:140552345	A=0.405	CRS-RFLP	61.9	Sense: TTTTTAGTAGAGACAGGGTTTTGCC Antisense: ACTCTACGCCAGACAATATGCTATG

a Minor allele frequency, based on the Chinese Han population data of the international HapMap project

In addition, the restriction enzyme AvaII and NsiI (Fermentas, Canada) were used for genotyping of rs10463297 and rs801460 respectively. The digestion patterns were separated by 3% agarose gel electrophoresis with ethidium bromide. The wild-type genotype of rs10463297 TT produced one 483 bp fragment; the TC genotype (heterozygote) produced 483, 317 and 166 bp fragments; CC genotype (variant homozygote) produced 317 and 166 bp fragments. The wild-type genotype of rs801460 GG produced one 294 bp fragment; the GA genotype produced 294, 271 and 23 bp fragments; AA genotype produced 271 and 23 bp fragments. All analyses were performed without knowledge of the case or control status for quality control. 10% of the study populations were randomly selected to confirm the genotyping results by different persons. In addition, a 10% random sample was also examined by direct sequencing (BGI Sequencing, Beijing). The results of confirmation were found to be 100% concordant.

### Real-time reverse transcription PCR analysis of SRA mRNA expression levels in plasma

To explore the effects of different genotypes of rs10463297 on the SRA mRNA expression, the relative levels of SRA mRNA was examine using SYBR-Green real-time quantitative PCR method in 82 samples obtained from cancer-free controls whose genotypic data were anonymous. Total RNA was isolated from blood plasma samples using TRIzol LS Reagent (Ambion). Then cDNA was synthesized with Primescript RT Reagent (Takara, Japan). The SRA primers used for quantitative real-time PCR were as follows: forward primer 5′- CAAGCGGAAGTGGAGATGGCGGAGC-3′ and reverse primer 5′- GCGAAGTGTGTAGGGAGCGGAGGCG-3′. For β-actin, as an internal reference gene, the primers used were 5′-AGAAAATCTGGCACCACACC-3′ and 5′-TAGCACAGCCTGGATAGCAA-3′ [35]. Amplification reactions were performed in a final volume of 20 μl containing10.0 μl Master mix, 150 ng cDNA, 1 μl primers. The reaction conditions of Real-time PCR were set at 95°C for 30s, followed by 40 cycles at 95°C for 5 s and 60°C for 30 s. All procedures were performed in triplicate. The expression of individual SRA mRNA expression measurements was calculated relative to expression of β-actin using the2^−ΔCT^ method.

### Statistical analysis

Our case-control study ample size was estimated with the PSAA 11.0 software, and calculate the sample size of gene-environment interaction was calculated by Quanto software under dominant inheritance model (http://biostats.usc.edu/cgi-bin/DownloadQuanto.pl). Hardy–Weinberg equilibrium (HWE) was tested by using a goodness-of-fi χ^2^-test to compare the observed genotype frequencies with the expected ones among the cancer-free control subjects. The differences in the distributions of age, reproductive variables, as well as the SNPs genotype frequencies between BC cases and controls, were appraised by using student's t test (for continuous variables) and Chi-squared (χ^2^) test (for categorical variables). Unconditional logistic regression models were used to evaluate the association between case–control status and each SNP by the odds ratio (OR) and its corresponding 95% confidence interval (95%CI), with adjustments for age, age at menarche, status of menopausal, number of pregnancy, number of abortion, breast-feeding history for born baby, family history of BC in first-degree relatives. Furthermore, the data were stratified by age and reproductive factors to evaluate the stratum variable-related ORs among various SRA SNPs. Multifactor Dimensionality Reduction (MDR) method was also performed to assess the potential interactions among gene–reproductive factors. Haplotype analysis was conducted using the online SHEsis (http://analysis.bio-x.cn/myAnalysis.php). For all significant genetic effects observed in our study, the false positive associations were calculated by FPRP (false positive report probability) with prior probabilities of 0.001, 0.01, 0.1, and 0.25. The OR was set at 1.5 under dominant genetic model, and a probability < 0.5 was considered as noteworthy. Statistical analysis was performed by using SPSS 16.0 software package (SPSS Inc., Chicago, IL, USA) and SAS 9.2 software package. A two-sided P value less than 0.05 was considered as the significant level.
